# Voices from the Community: New insights into costs of dementia from persons with lived experience, public health leaders and health care providers

**DOI:** 10.1002/alz70860_101354

**Published:** 2025-12-23

**Authors:** Lycia Tramujas Vasconcellos Neumann, Matthew Baumgart, Lauren Stratton, Chelsea Renee Kline, Kerry Finnegan, Ashley Kuzmik, Maria C. Carrillo

**Affiliations:** ^1^ Alzheimer's Association, Chicago, IL, USA; ^2^ Alzheimer's Association, District of Columbia, DC, USA

## Abstract

**Background:**

The economic impact of dementia on individuals, families, communities, systems, and societies has not been fully assessed for integration into estimates of dementia costs. Through a joint effort of the University of Southern California and the Alzheimer's Association, a Family and Persons Living with Dementia (FPLWD) Panel and a Public Health and Care Expert (PHCE) Panel were established to identify direct, indirect, and intangible dementia costs. Insights from the panels support comprehensive estimates of dementia costs produced by the United States Costs of Dementia Model (USCDM).

**Methods:**

Panel members were recruited through the Alzheimer's Association chapters, engagement and advisory groups, and national network to ensure panels included diverse lived experiences of individuals living with dementia, caregivers, care partners, the public health community, and professionals involved in dementia care and support. In quarterly online meetings of approximately 2 hours, panel members ‐ 10 FPLWD and 11 PHCE ‐ discuss costs associated with dementia diagnosis, treatment, and care guided by prompt questions. Meetings are recorded, and transcripts are thematically analyzed.

**Results:**

Participants of both the FPLWD and PHCE panels have engaged actively in the discussions and brought complementary perspectives about the economic impact of dementia in the US. Initial findings indicate consistency between panels on the most pressing direct costs associated with dementia, such as medical expenses, home modifications, and transportation. In both panels, members also identified indirect costs associated with work‐related changes, families' income loss, and those related to the impact on caregivers’ health and well‐being. From their different perspectives, each panel shared information about potential hidden costs that affect individuals, families, and systems, such as those related to identity and social impact, financial management, and family medical leave absence.

**Conclusions:**

Individuals with lived experience and public health and care experts bring invaluable perspectives to a comprehensive understanding of the various ways dementia affects individuals, families, communities, organizations, systems, and society. While several of these findings align with existing knowledge about the costs associated with dementia, many insights shed light on novel aspects to measure when quantifying the economic impact of dementia within the country.

